# Potentially Infectious Novel Hepatitis A Virus Strains Detected in Selected Treated Wastewater Discharge Sources, South Africa

**DOI:** 10.3390/v12121468

**Published:** 2020-12-19

**Authors:** Saïd Rachida, Maureen Beatrice Taylor

**Affiliations:** 1 Department of Medical Virology, Faculty of Health Sciences, University of Pretoria, Private Bag X323, Gezina, 0031 Pretoria, South Africa; achidasaid@gmail.com; 2 School of Health Systems and Public Health, Faculty of Health Sciences, University of Pretoria, 0002 Pretoria, South Africa; 3 National Health Laboratory Service, Tshwane Academic Division, 0002 Pretoria, South Africa

**Keywords:** hepatitis A virus, viability PCR, wastewater discharge, South Africa, novel HAV variant

## Abstract

Hepatitis A virus (HAV) is a waterborne pathogen of public health importance. In South Africa (SA), unique HAV subgenotype IB strains have been detected in surface and wastewater samples, as well as on fresh produce at the point of retail. However, due to the use of molecular-based assays, the infectivity of the detected strains was unknown. Considering the potential shift of HAV endemicity from high to intermediate, which could increase the risk of severe symptomatic disease, this study investigated the identity of HAV strains detected before and after viability treatment of selected wastewater discharge samples. For one year, 118 samples consisting of sewage, treated wastewater discharge and downstream dam water were collected from five wastewater treatment plants (WWTP 1, 2, 3, 4 and 5). Unique HAV IB strains were detected in samples from all five WWTPs, with 11 of these strains carrying amino acid mutations at the immunodominant and neutralisation epitopes. A quasispecies dynamic of HAV has also been detected in sewage samples. The subsequent application of viability PCR revealed that potentially infectious HAV strains were discharged from WWTP 1, 2, 4 and 5 into the dam. Therefore, there is a potential risk of HAV exposure to communities using water sources downstream the WWTPs.

## 1. Introduction

Hepatitis A virus (HAV), a leading cause of acute hepatitis worldwide [1], is an important waterborne pathogen with significant public health impact [2,3]. The HAV virion, shed in the faeces of infected individuals, is resistant to wastewater treatment processes [4] and can remain infectious in untreated and treated wastewater for days to months [5]. Consequently, rivers receiving HAV-contaminated wastewater discharge could in turn become polluted, exposing users to potential infection with HAV [6]. Given that all infected individuals, be they symptomatic or not, shed the virus in faeces [7], the analysis of sewage, wastewater and surface water can provide a more accurate estimation of the HAV strains circulating in a specific community. In addition, this surveillance system could serve as an early warning system of potential outbreaks [8].

Hepatitis A virus is the type species of the genus *Hepatovirus*, classified within the *Picornaviridae* family [9]. The genome of HAV is a positive-sense single stranded non-segmented RNA molecule of approximately 7500 nucleotides that has a single open reading frame (ORF) [1,9]. The translated polyprotein is cleaved into structural and non-structural proteins [1,9]. Six geographically distinct HAV genotypes have been identified by nucleotide sequence analysis of the genomic region encoding the VP1 capsid protein [10]. These genotypes infect humans (genotypes I, II and III) and non-human primates (genotypes IV, V and VI) [10]. The simian HAV strains have a unique VP3/VP1 junction that distinguishes them from human HAV [6]. The stable antigenic structure of HAV has yet to be completely characterised, but findings suggest that it is defined by four epitopes [11,12,13,14]. Certain amino acid residues of the VP1 protein contribute to the immunodominant antigenic site and the neutralisation epitope, also known as the glycophorin A binding site. It has also been shown that replacement of a single amino acid residue at these epitopes could be enough to confer resistance to antibody neutralisation [12,13,15].

In South Africa (SA), HAV has been detected in clinical [16] and environmental samples, namely sewage, treated wastewater discharge, various water sources and on fresh produce at the point of retail [17,18,19,20]. The South African (SAn) strains predominantly belong to subgenotype IB but carry unique mutations which distinguish them from IB strains detected in other regions worldwide [21,22]. A novel HAV strain as well as a potential vaccine escape mutant have also been detected in separate single irrigation water samples from two different provinces in SA [22,23]. However, as the viruses were detected by molecular-based assays it is not known whether the strains were potentially infectious or not. Recently, molecular-based assays that can distinguish potentially infectious from non-infectious viruses have been developed [24,25]. The pre-treatment of samples with intercalating dyes, e.g., propidium monoazide (PMA) or ethidium monoazide (EMA), prior to nucleic acid extraction allowed the successful detection of potentially infectious HAV particles in environmental samples [3,26,27]. The pre-treatment and downstream PCR are called viability treatment and viability PCR (vPCR), respectively [24]. The genetic characterisation of HAV strains following viability treatment could be more valuable from a public health perspective, defining potentially infectious virus genotypes in the environment.

Hepatitis A was considered to be hyperendemic in SA [16]. However, recent serological studies have shown that the endemicity could be shifting from high to intermediate levels [28,29], which may increase the risk of severe symptomatic disease in older individuals [6]. Therefore, the aim of this study was to determine the genetic identity of HAV strains detected before and after viability treatment of SAn selected wastewater discharge samples.

## 2. Materials and Methods

### 2.1. Sample Collection and Processing

From April 2015 to March 2016, a comprehensive water sampling programme was initiated to establish the contribution of wastewater discharge from wastewater treatment plants (WWTPs) to contamination in the surface catchment and source water, i.e., dam, for a large water treatment utility. Sewage inflow (1 L) and treated wastewater discharge (10 L) from five selected WWTPs (WWTP 1, 2, 3, 4 and 5) and dam water (10 L) samples were collected monthly for analysis. The samples were transported on ice to the laboratory and stored at 4 °C until processing.

Viruses were recovered and concentrated from the sewage influent, treated discharge and dam water samples to a final volume of 10 mL in phosphate buffered saline (PBS; pH 7.2, (Sigma-Aldrich Co., St Louis, MO, USA)) using previously optimised methods. A glass wool adsorption-elution method was used to recover the viruses from the 10 L treated wastewater discharge and dam water samples [20,30,31]. The recovered viruses were further concentrated by polyethylene glycol (PEG_8000_; Amresco, Solon, OH, USA)/sodium chloride (NaCl; Merck KGaA, Darmstadt, Germany) precipitation [32]. The PEG_8000_/NaCl precipitation method alone was used to concentrate viruses from the 1 L influent sewage samples. Aliquots (1 mL) of virus concentrates were stored at −20 °C until further investigation.

### 2.2. Initial HAV Screening by RT-qPCR

Total nucleic acid was extracted from the virus concentrate (1 mL) and eluted into 100 µL of nuclease-free water (Promega Corp. Madison, WI, USA) using the semi-automated NucliSENS^®^ EasyMAG^®^ platform (BioMérieux, Marcy l’Etoile, France), according to the manufacturer’s instructions. Prior to extraction, 5 × 10^4^ genome copies (gc) of mengovirus (MC_0_) were added to each aliquot of virus concentrate as a process control. Aliquots of the extracted nucleic acid were stored at −70 °C until further analyses.

Mengovirus was quantified using the mengo@ceeramTools™ Kit (Ceeram s.a.s, La Chappelle-Sur-Erdre, France) and an RNA standard [33]. Samples that tested negative for mengovirus were re-extracted and retested. Hepatitis A virus was detected and quantified using the hepatitisA@ceeramTools™ Kit (Ceeram s.a.s), which uses proprietary primers and probe, and a HAV DNA quantification standard (Ceeram s.a.s). The RT-qPCR assay was performed using 5 µL of nucleic acid in a 25 µL reaction. The RT step was performed at 45 °C for 10 min followed by initial denaturation at 95 °C for 10 min. Thereafter, amplification was performed with 45 cycles of 95 °C for 15 s and 60 °C for 45 s on the Lightcycler^®^ v2.0 (Roche Diagnostics GmbH, Mannheim, Germany). The hepatitisA@ceeramTools™ Kit includes an internal control (IC) that monitors amplification progress. In addition, a positive control, provided with the kit, and a negative control (nuclease-free water (Promega Corp.)) were included in every HAV RT-qPCR assay.

### 2.3. HAV Quantification by vPCR

Available HAV-positive virus concentrates were subjected to a viability treatment using a combination of PMA™ (Biotium, Fremont, CA, USA) and Tween^®^20 (Molecular Biology Grade, Promega Corp.). The protocol developed by Moreno et al. [26] was optimised and adapted for the selective quantification of infectious HAV in SAn river water samples [34]. The dyes PMA™ (Biotium) and EMA (Biotium) combined or not with the surfactants Triton^®^ X100 (Molecular Biology Grade, Promega Corp.) or Tween^®^20 (Promega Corp.) were tested. The best results were achieved by treating the samples with PMA and Tween^®^20 (Promega Corp.) prior to nucleic acid extraction [34].

A 20 mM PMA™ working solution, made by dissolving 1.0 mg of PMA™ in 98 µL of nuclease-free water (Promega Corp.), was used for the viability treatment. Tween^®^20 followed by PMA™ were added to 0.2 mL or 1 mL of HAV-positive virus concentrates at a final concentration of 0.5% and 50 µM, respectively. The resultant mixture was gently mixed by inversion three times, then incubated for 10 min in the dark at 25 °C on a microplate shaker (IIKA^®^ Schüttler Microplate Shaker Model MTS 4 (IKA, Janke and Kunkel, GmbH, Mannheim, Germany)) set to 150 rpm. Thereafter, photoactivation of the mixture was performed for 15 min on a PMA-Lite™ LED Photolysis Device (Biotium) following the manufacturer’s instructions. Total nucleic acid was extracted from the 0.2 mL or 1 mL treated virus concentrates and eluted into 50 µL or 100 µL of nuclease-free water (Promega Corp.), respectively. The extraction was performed on the semi-automated NucliSENS^®^ EasyMAG^®^ platform (BioMérieux), according to the manufacturer’s instructions. The quantification of HAV was performed as described for the initial screening

### 2.4. Partial Genome Amplification

Hepatitis A virus strains detected during initial screening were further characterised using the VP1 and VP1/P2B genomic regions. Due to sample availability, HAV strains from a sewage (FR1) sample and a treated wastewater discharge (FE8) sample from WWTP4, which tested positive for HAV after viability treatment, were also selected for genotyping.

Complementary DNA was synthesised using 10 µL of nucleic acid extracted from the HAV-positive virus concentrates. The synthesis was performed using the Protoscript^®^ II Reverse Transcriptase (New England Biolabs^®^, Ipswich, MA, USA) with minor modifications to the manufacturer’s protocol, i.e., the 20 µL reaction mixture contained 30 µM of random hexamers (Roche Diagnostics), 50 U Protoscript^®^ II Reverse Transcriptase and 20 U Protoscript^®^ II RNase inhibitor. The synthesised cDNA was used immediately for PCR amplification of the VP1 and VP1/P2B genomic regions.

The entire VP1 region (900 nt) was amplified in two rounds of conventional PCR using the EmeraldAmp^®^ MAX HS PCR Master Mix (Takara Bio Inc., Shiga, Japan) and published primers (Table 1). In the first round of amplification, the 50 µL reaction mix contained 5 µL of cDNA, 25 µL of EmeraldAmp^®^ MAX HS PCR Master Mix (2× Premix) (Takara Bio Inc.), 1 µL of HAV1 forward primer (0.2 µM), 1 µL of HAV2 reverse primer (0.2 µM) and 18 µL of nuclease-free water (Promega Corp.). The cycling conditions were 30 cycles of 98 °C for 10 s, 50 °C for 30 s, 72 °C for 1 min, and, final extension at 72 °C for 5 min. The second round PCR was performed using the 2172P and 3125N primers (Table 1) and 1 µL of the completed first round PCR. The amplification conditions of the second round PCR were the same as the first round except for the annealing temperature, which was decreased from 50 °C to 48 °C.

In addition, a 350 nt region of the VP1/P2B junction was also amplified in two rounds of conventional PCR. The 50 µL reaction mix included 5 µL of cDNA, 25 µL of EmeraldAmp^®^ MAX HS PCR Master Mix (2× Premix) (Takara Bio Inc.), 1 µL of 2870P (0.2 µM), 1 µL of 3381N (0.2 µM) and 18 µL of nuclease-free water (Promega Corp.). The first round PCR conditions were: 30 cycles of 98 °C for 10 s, 45 °C for 45 s, 72 °C for 30 s, and, final extension at 72 °C for 5 min. The primers 2896P and 3289N (Table 1) together with 1 µL of the completed first round PCR were used for the second round of amplification of the VP1/P2B junction. The amplification conditions were the same as the second round PCR conditions used for amplification of the VP1 region.

### 2.5. Cloning and Sequencing

Amplicons were analysed using 2% agarose gel (SeaKem® LE Agarose, Lonza, Basel, Switzerland) electrophoresis. Visualisation was achieved by ethidium bromide staining, followed by UV illumination. Amplicons were purified using the Zymogen DNA Clean & Concentrator-25™ Kit (Zymo Research, Irvine, CA, USA).

The amplified segments were cloned using the CloneJET PCR cloning Kit (Thermo Fisher Scientific, Nunc A/S, Roskilde, Denmark) as per the manufacturer’s instructions. After transformation, a minimum of seven colonies were randomly selected for colony PCR, which was achieved using the One Taq^®^ Quick-Load^®^ 2X Master Mix with Standard Buffer (New England Biolabs) and the pJET1.2 forward and reverse primers. At least five positive clones were purified using the Zymogen DNA Clean & Concentrator-25™ Kit (Zymo Research) and selected for Sanger sequencing.

The purified amplicon was sequenced in both directions on an ABI 3130 automated analyser (Applied Biosystems, Foster City, CA, USA) using the ABI Prism BigDye^®^ Terminator v3.1 Cycle sequencing Kit. The 20 µL sequencing reaction contained the following: 3 µL of BigDye™ Terminator v3.1 5X Sequencing Buffer, 1 µL of BigDye™ Terminator v3.1 Ready Reaction Mix, 1 µL of pJET forward or reverse primer (3.2 µM), 3 µL of clean PCR product and 12 µL of nuclease-free water (Promega Corp.).

### 2.6. Phylogenetic Analysis

Raw sequences were analysed using the Sequencher™ v4.10.1 (Gene Codes Corporation, Ann Arbor, MI, USA) and the BioEdit Sequence Alignment Editor (v6.0.5.2). The identity of edited sequences was verified using the Basic Local Alignment Search Tool (BLAST) program [36] of GenBank and the Hepatitis A Virus Genotyping tool v1.0 (available at https://www.rivm.nl/mpf/typingtool/hav/job/1899775064/). Verified nucleotide sequences were submitted to GenBank under the following accession numbers: MT380563 to MT380626 and MT721458 to MT721725 for the VP1 region; MT380641 to MT380709 and MT721175 to MT721457 for the VP1/P2B genomic region. The nucleotide sequence of strains detected after viability treatment were assigned the accession numbers MT380627 to MT380640 and MT380710 to MT380722 for the VP1 and VP1/P2B genomic regions, respectively.

Multiple alignments of the verified nucleotide sequences, together with reference sequences of each genotype of HAV and closely related sequences from GenBank (Table 2), were created in MAFFT v7.110 (http://mafft.cbrc.jp/alignment/server/). Nucleotide sequence data of HAV strains previously characterised from clinical (Table 2) and water sources (Table 2) that were available in GenBank were also included in the alignment. These strains were detected in specimens and samples collected from SA, Swaziland, Kenya and Tanzania. Aligned nucleotide sequences were checked manually, translated into protein in BioEdit Sequence Alignment Editor (v6.0.5.2) and compared to HAV reference strains in order to detect any novel or previously described amino acid mutation. The position of recorded amino acid changes was relative to HAV HM175 strain (M14707).

The evolutionary history of detected HAV strains was inferred using the Neighbour-Joining method [37] in MEGA X [38]. The evolutionary distance between the detected strains and previously characterised HAV strains was computed using the Kimura two-parameter method [39]. The constructed phylogenetic trees were assessed by bootstrap analyses (1000 replicates) [40] and a value of 70% was considered significant.

## 3. Results

### 3.1. Screening

A total of 118 samples, collected from WWTPs 1, 2, 3, 4 and 5, were screened for HAV. Based on the quality controls, i.e., positive, negative and IC, the detection assays were considered valid. Hepatitis A virus was detected in samples from all five WWTPs with 80% (43/54) of sewage and 83% (43/52) of treated wastewater discharge testing positive, while no virus was detected in the 12 dam water samples (Table 3). Viral titres ranged from 1.34 × 10^5^ to 3.70 × 10^10^ gc/L of sewage and from 4.74 × 10^3^ to 3.39 × 10^7^ gc/L of treated wastewater discharge. Of the 79 HAV-positive samples which were subjected to viability treatment, potentially infectious HAV was quantified from the virus concentrates of 81% (30/37) of sewage (viral titres ranged from 7.57 × 10^2^ to 2.16 × 10^6^ gc/L) and 90% (38/42) of treated wastewater discharge (viral titres ranged from 7.83 × 10^1^ to 3.34 × 10^4^ gc/L) samples (Table 3). Potentially infectious HAV was discharged from WWTPs 1, 2, 4 and 5 into the dam or rivers feeding into the dam, while no potentially infectious HAV was discharged from WWTP 3 (Table 3).

### 3.2. Nucleotide Sequence and Phylogenetic Analyses

Following preliminary screening, HAV could be genotyped by nucleotide sequence analysis of the VP1 and VP1/P2B genomic regions from 79% (68/86) and 84% (72/86) of wastewater samples, respectively. Pairwise analyses, performed over the VP1 and VP1/P2B genomic regions, showed that the strains detected in this study were 90.3–95.6% and at least 94.7% identical to the HM175 strain at the nucleotide and amino acid levels, respectively.

**Figure 1:**
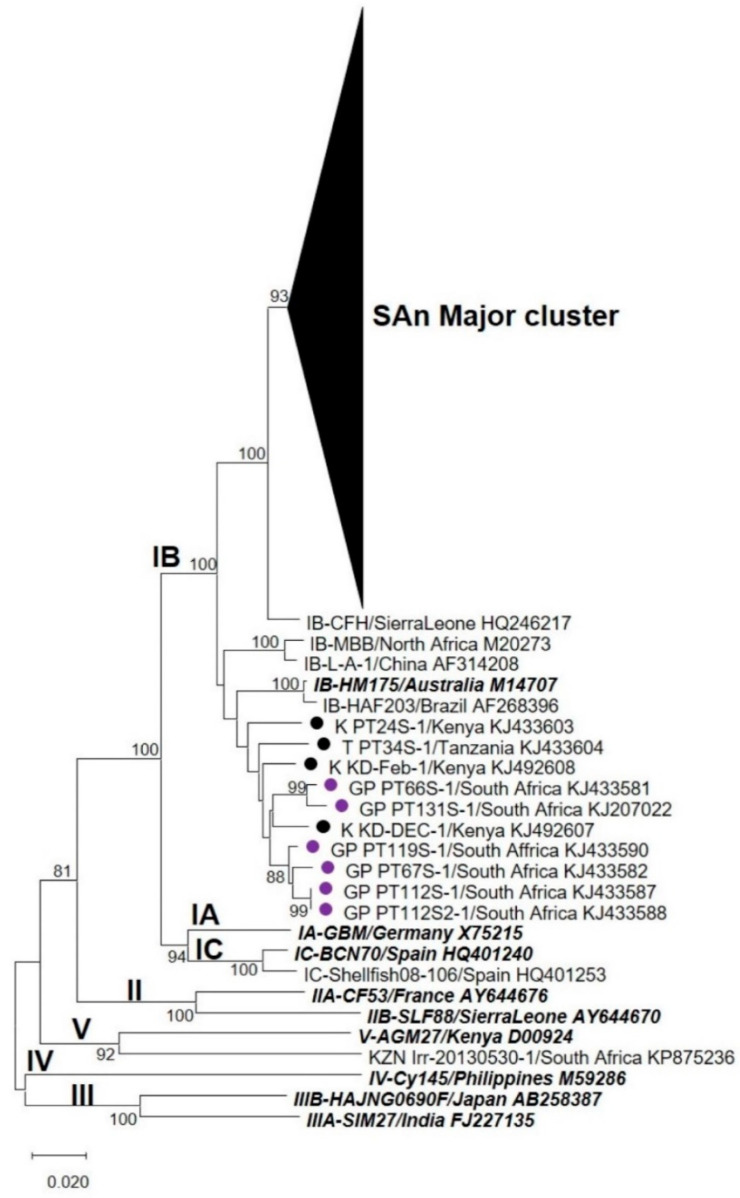
Phylogenetic analysis targeting the VP1 region of hepatitis A virus (HAV) strains detected in samples from all five wastewater treatment plants (WWTPs). Previously characterised HAV strains from clinical specimens in Gauteng (●) and from other countries (●) are indicated. The names of type strains for each genotype and subgenotype are bolded and italicised. The neighbour-joining tree was constructed using the Kimura two-parameter model. Bootstrap values greater or equal to 70% are shown at the nodes. The nucleotide sequence alignment used to infer the analysis is provided as Supplementary file 1 (Fasta file).

**Figure 2:**
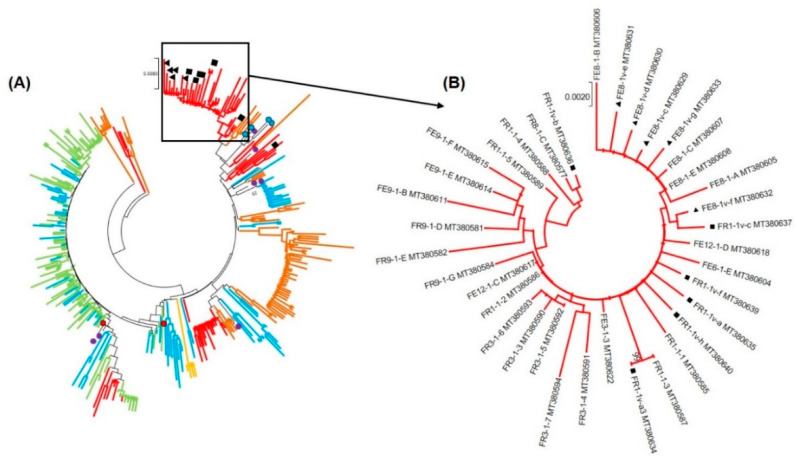
Phylogenetic analysis targeting the VP1 region of hepatitis A virus (HAV) strains detected in samples from all five WWTPs. (**A**) HAV strains of the “SAn Major cluster” are highlighted. For better visualisation, branches are coloured blue, green, orange, red and brown to represent virus lineages from WWTP1, 2, 3, 4 and 5, respectively. Previously characterised HAV strains from wastewater (●) and clinical (●) sources in Gauteng and from Swaziland (●) are indicated. HAV strains detected after viability treatment are indicated with ■ (sewage) and ▲ (treated discharge). (**B**) HAV strains characterised from WWTP4 before and after viability treatment are highlighted. The neighbour-joining trees were constructed using the Kimura two-parameter model. Bootstrap values greater or equal to 70% are shown at the nodes.

**Figure 3:**
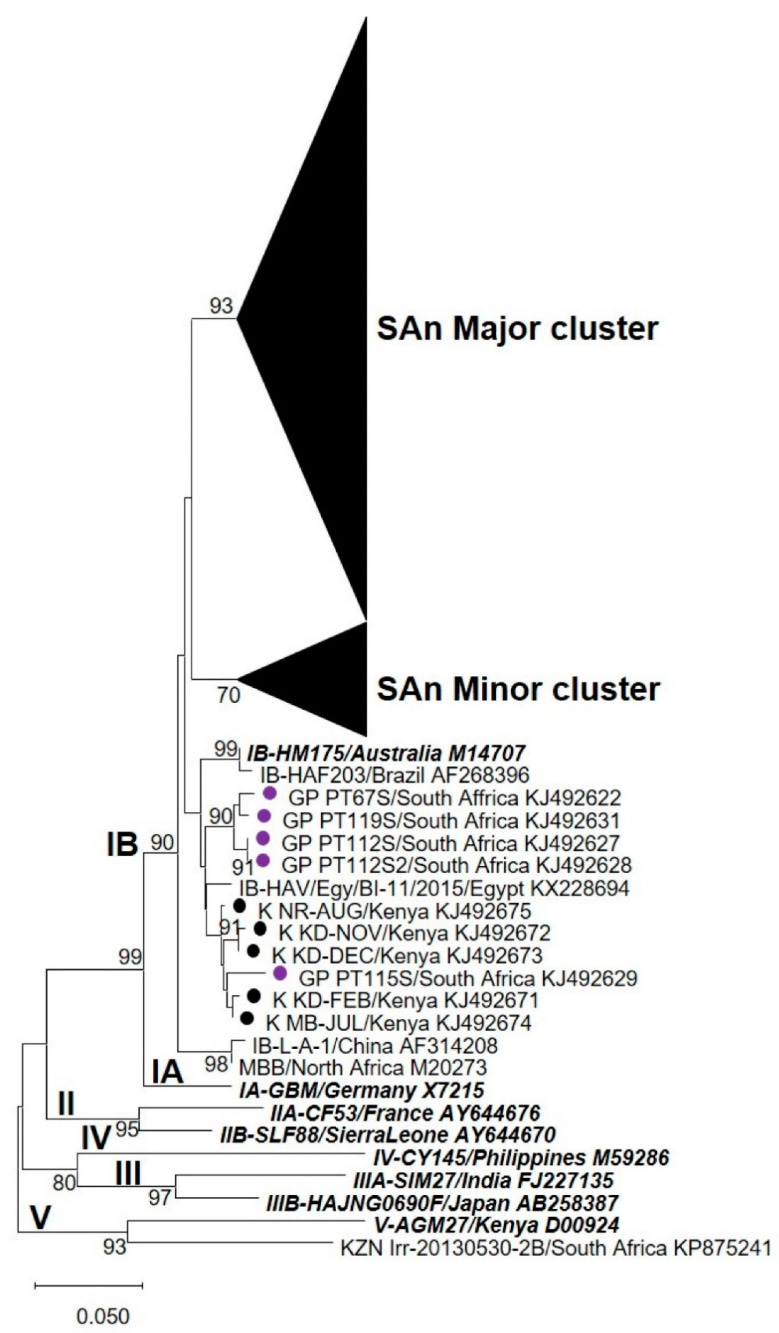
Phylogenetic analysis targeting the VP1/P2B junction of hepatitis A virus (HAV) strains detected in samples from all five wastewater treatment plants (WWTPs). Previously characterised HAV strains from clinical sources in Gauteng (●) and from other countries (●) are indicated. The names of type strains for each genotype and subgenotype are bolded and italicised. The neighbour-joining tree was constructed using the Kimura two-parameter model. Bootstrap values greater or equal to 70% are shown at the nodes. The nucleotide sequence alignment used to infer the analysis is provided as Supplementary file 2 (Fasta file).

**Figure 4:**
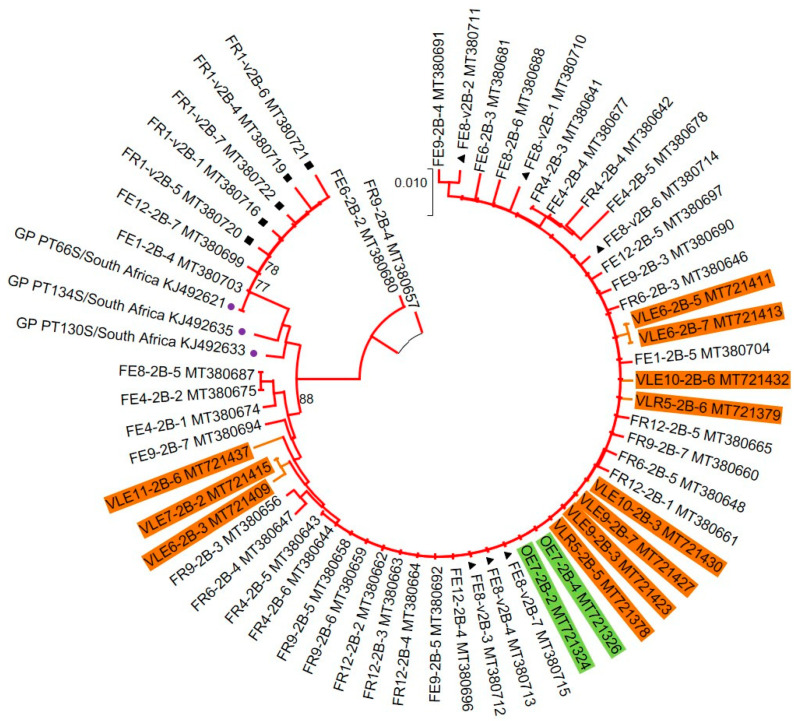
Phylogenetic analysis targeting the VP1/P2B junction of hepatitis A virus (HAV) strains detected in samples from all five wastewater treatment plants (WWTPs)—the tree highlights strains from the “SAn Minor cluster”. For better visualisation, branches are coloured red and brown to represent virus lineages from WWTP4 and 5, respectively. HAV strains detected in samples from WWTP2 (green) and WWTP5 (brown) are highlighted. Viruses detected after viability treatment are indicated with ■ (sewage) and ▲ (treated discharge). Previously characterised HAV strains from clinical sources in Gauteng (●) are indicated. The neighbour-joining tree was constructed using the Kimura two-parameter model. Bootstrap values greater or equal to 70% are shown at the nodes.

Phylogenetic analyses, performed over the VP1 region, revealed that HAV strains characterised from all five WWTPs formed a unique cluster (“SAn Major cluster”) within genotype IB (Figure 1). The “SAn Major cluster” groups the strains detected in this study (highlighted in Figure 2A) together with HAV strains previously characterised from water and clinical sources collected in Gauteng (Figure 2A). Within the “SAn Major cluster”, HAV strains tend to cluster by WWTP except for a few strains from WWTP1 and 2 (alternating blue and green colours in Figure 2A). Hepatitis A virus strains from Swaziland (SZ_PT126S and SZ_PT29S) also cluster within the “SAn Major cluster” (Figure 2A), while strains from Kenya (K_PT24S, K_KD-Feb and K_KD-Dec) and Tanzania (T_PT34S) do not (Figure 1). Phylogenetic analyses performed over the VP1/P2B junction also recorded a “SAn Major cluster” for HAV strains characterised from samples collected from all five WWTPs (Figure 3). However, several HAV strains, characterised from samples collected from WWTPs 2, 4 and 5, are grouped within the “SAn Minor cluster” but are still within subgenotype IB (Figure 3). The minor cluster includes HAV strains previously characterised from clinical specimens in Gauteng (Figure 4), one of which (GP PT66S, [KJ492621]) is grouped outside the major cluster during analysis performed over the VP1 region (Figure 1).

Hepatitis A virus strains that were detected after viability treatment of samples from WWTP4 are grouped within the “SAn Major cluster” (indicated with ■ (sewage) and ▲ (treated discharge) in Figure 2). However, analyses based on the VP1/P2B junction revealed that these strains are included within the “SAn Minor cluster” (Figure 4).

### 3.3. Amino Acid Analysis

**Figure 5:**
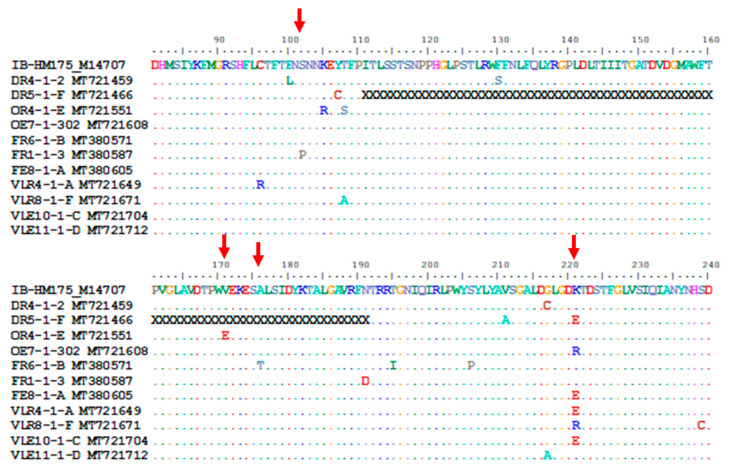
Alignment of the deduced amino acid sequences of the VP1 region of the HM175 strain and hepatitis A virus (HAV) strains carrying mutations at immunodominant and neutralisation epitopes. Only amino acid position 81 to 240 is shown. Conserved sites, substitutions and deletions are represented by dots, single-letter abbreviation and the letter “X”, respectively. The red arrows point to the positions of the immunodominant (102, 171 and 176) and neutralisation (221) epitopes. The complete protein alignment from position 1 to 300 is provided as Supplementary files 3 (graphic view) and 4 (Fasta file).

Amino acid sequences of the VP1 region revealed that 94% of the sequences, obtained from sewage and treated discharge samples, carry the R298K amino acid change (Supplementary file 1) as recorded for HAV IIIA (Sim27, FJ227135) and IIIB (HAJNG0690F, AB258387) strains. A total of 11 HAV strains carrying amino acid changes at the immunodominant (S102, V171 and A176) and neutralisation (K221) epitopes were detected in sewage and treated discharge samples (Table 4). Amino acid substitutions have also been recorded at G217, which is known to influence the neutralisation epitope. The majority (6/11) of strains have a mutation at position 221 (K221E or K221R) (Table 4) (Figure 5).

**Figure 6:**
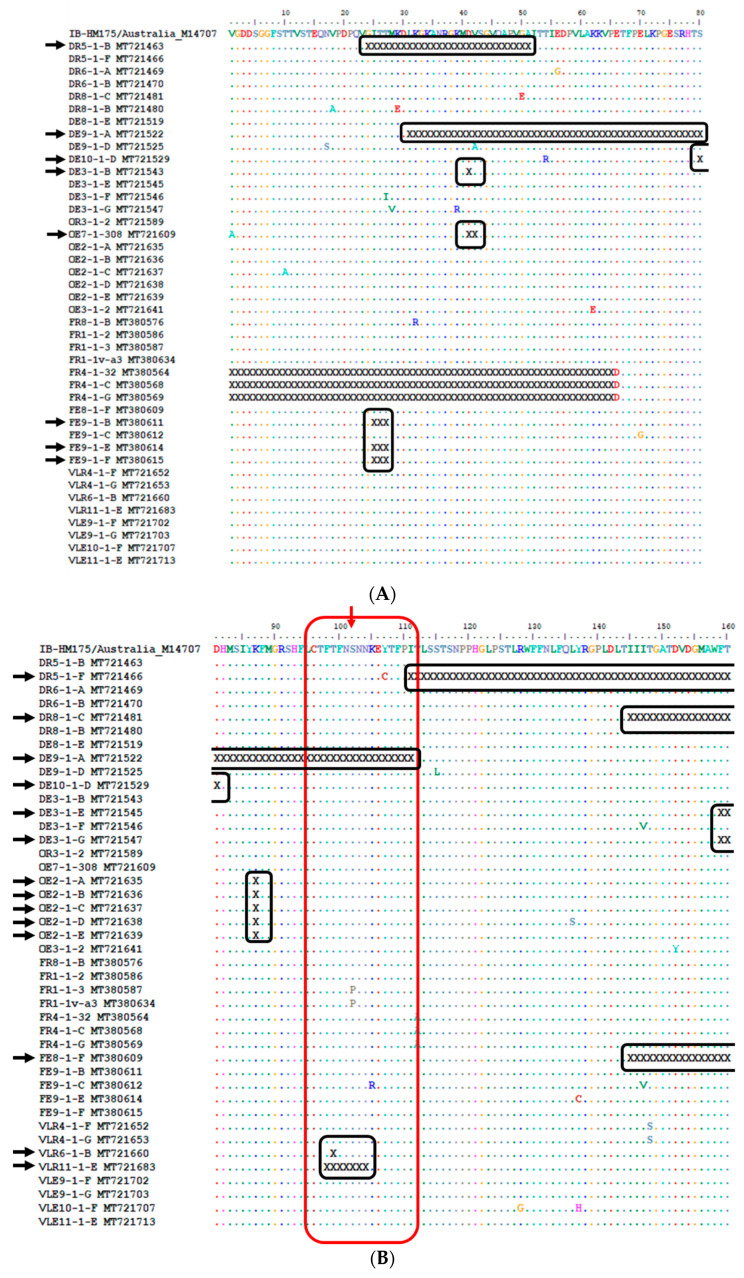

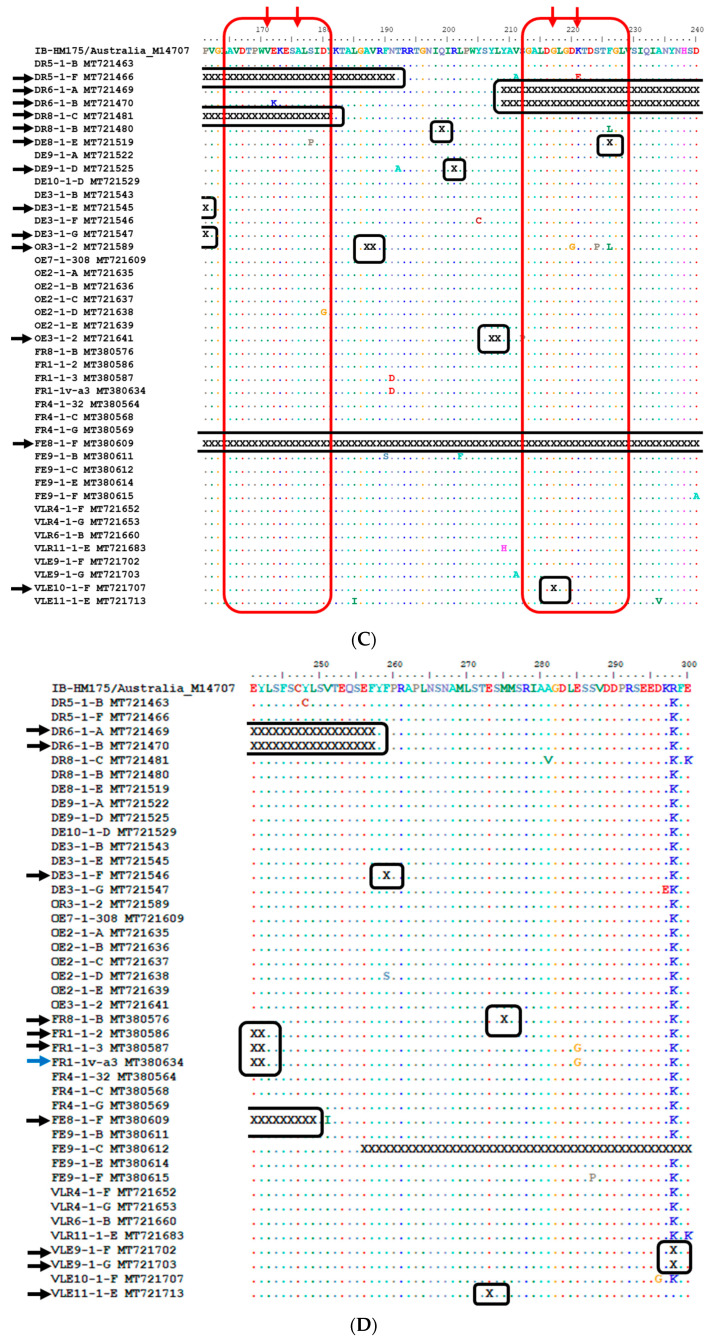
Alignment of the deduced amino acid sequences of the VP1 region of the HM175 strain and hepatitis A virus (HAV) strains carrying in-frame deletions (**A**–**D**). Conserved sites, substitutions and deletions are represented by dots, single-letter abbreviation and the letter “X”, respectively. The red arrows and blocks highlight amino acid change at epitopes 102 (**B**), 171, 176, 217 and 221 (**C**), and their surroundings. The closed black blocks highlight in-frame deletions within a subfigure. The open black blocks highlight in-frame deletions that span between two or more subfigures. The black arrows point to HAV strains with in-frame deletion(s). The blue arrow points to a HAV strain with in-frame deletion, which was characterised after viability treatment. The sequence alignment used to construct Figure 6 is provided as Supplementary file 5 (Fasta file).

The amino acid analysis of the VP1 region also recorded in-frame deletions (Figure 6). The size of these deletions ranged from one amino acid (DE3-1-B MT721543 in Figure 6A) to 105 (FE8-1-F MT380609 in Figure 6B–D) amino acids, and they are mostly located within the sequences of strains characterised from sewage and treated discharge samples (Figure 6). Some of these in-frame deletions include the epitope and surrounding amino acids (Figure 6B,C).

**Figure 7:**
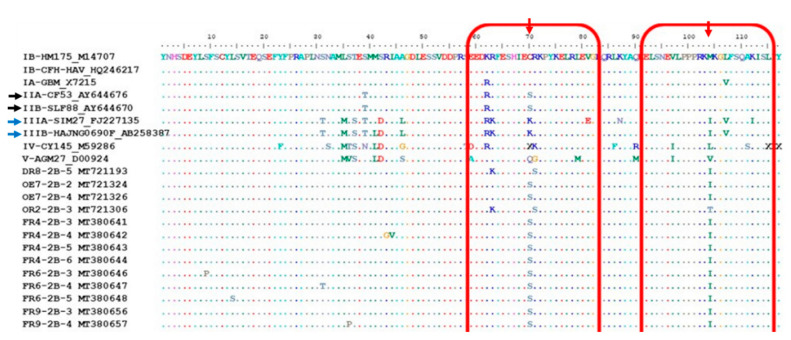
Alignment of the deduced amino acid sequences of the VP1/P2B junction of the HM175 strain and 13 out of the 47 hepatitis A virus (HAV) strains carrying the C70S and/or M104I changes. Conserved sites, substitutions and deletions are represented by dots, single-letter abbreviation and the letter “X”, respectively. The red arrows and blocks highlight amino acid change at position 70 and 104, and their surroundings. The black arrows point to HAV genotype II reference strains that carry the C70S change. The blue arrows point to HAV genotype III reference strains that carry the M104I change. The protein alignment containing all 47 HAV strains is provided as Supplementary files 6 (graphic view) and 7 (Fasta file).

The R63K and R71S were the most abundant (87% of sequences) amino acid changes recorded during analysis of the VP1/P2B junction (Supplementary file 2). The R63K change corresponds to the R298K change recorded for sequences of the VP1 region. In addition, HAV strains carrying the C70S and/or M104I amino acid changes (indicated with red arrows in Figure 7) were recorded. The majority (76%) of these strains are grouped within the “SAn Minor cluster” (Figure 3 and Figure 4). The HAV reference strains of subgenotypes IIA (CF53, AY644676) and IIB (SLF88, AY644670) (indicated with black arrows in Figure 7) carry the C70S change, while the M104I change is present in the sequences of HAV IIIA (Sim27) and IIIB (HAJNG0690F) strains (indicated with blue arrows in Figure 7). In-frame deletions, ranging from one to four amino acids, were also recorded.

Potentially infectious HAV was detected in the 11 samples, from which possible antigenic escape variants of the virus were identified. Analysis of the amino acid sequences of HAV strains detected after viability treatment of the FR1 and FE8 wastewater samples from WWTP4 confirmed the presence of the most frequently detected amino acid changes before viability treatment (Table 5). In addition, a two amino acid in-frame deletion and strains carrying the S102 and G217 changes have been recorded in the FR1 sample after viability treatment (Table 5).

## 4. Discussion

The present study aimed to investigate the genetic identity of potentially infectious HAV strains detected in selected SAn wastewater sources. The results of the study revealed that the surveillance of wastewater samples, using a combination of vPCR, cloning, Sanger sequencing and phylogenies produced from the VP1 and VP1/P2B genomic regions, enabled the characterisation of unique HAV IB variants circulating within a SAn community.

This is the first report of the quasispecies dynamic of HAV in sewage samples. Hepatitis A virus strains with large in-frame deletions (up to 105 amino acids) were detected in sewage samples. An in vitro study suggested that in-frame deletions could be an adaptation mechanism adopted by HAV in the presence of new environmental conditions [15]. The in-frame deletions detected in the present study are located around the immunodominant and neutralisation epitopes and suggest the potential emergence of antigenic escape mutants. The structural constraints of the HAV capsid suggest that these deletions arose from immune selection pressure present in the community. South Africa has a large immunocompromised population with approximately 7.97 million people infected with HIV [41]. Previous studies have shown that incomplete vaccination within an immunocompromised population could increase the probability of the emergence of antigenic escape mutants [13,42]. However, evidence for the emergence of new variants of HAV has been provided in both vaccinated and unvaccinated patients [42]. Even though the HAV vaccine is not part of the national expanded program of immunisation, antigenic escape mutants can still emerge in the SAn community, as evidenced by the characterisation of HAV strains with amino acid changes at the immunodominant and neutralisation epitopes.

Hepatitis A virus strains, carrying mutations at the immunodominant and neutralisation epitopes, have been detected in sewage and treated wastewater discharge samples. It has been shown that HAV strains with amino acid changes at the immunodominant site (S102, V171 and A176) have lower fitness compared to wild-type strains [15]. This could explain why, in the present study, HAV strains with amino acid changes at positions 102, 171 and 176 were only detected in sewage samples (Table 4). On the other hand, HAV strains carrying amino acid change at position 217 have similar fitness to wild-type HAV and could potentially affect antibody binding at the K221 epitope [15]. This could explain the detection of HAV strains carrying amino acid changes at G217 and K221 in sewage and treated wastewater discharge. The fact that these strains were detected from treated discharge samples from which potentially infectious HAV strains were quantified is a cause for concern.

The results of pairwise and phylogenetic analyses indicated that IB is the only subgenotype present in the sampling region. Hepatitis A virus strains detected in the sampling region showed uniqueness to the population serviced by the treatment work investigated, but a close relationship to HAV strains previously characterised from clinical sources in Gauteng. Of importance are the HAV strains from WWTP2, 4 and 5 that formed the “SAn Minor cluster” together with three strains from clinical cases in Gauteng (Figure 4). Strains belonging to the minor cluster were detected before and after viability treatment and carried the same amino acid changes (C70S and M104I over the VP1/P2B genomic junction), suggesting that they might have originated from a group of people with common risk factors or exposure to a common source of HAV. Given that the shedding of HAV peaks during the incubation period, genetic analysis of HAV strains from wastewaters could allow the early detection of an outbreak, as shown by previous studies [8]. As clinical data from the community inhabiting the sampling region were not collected, it was not possible to confirm if these strains originated from an outbreak or sporadic cases.

The study was mostly limited by the few nucleotide sequences of HAV strains obtained after viability treatment. The production of a higher number of nucleotide sequences after viability treatment could have further increased the probability of detecting infectious antigenic escape variants of HAV in treated wastewater discharge. Despite the low number of nucleotide sequences obtained after viability treatment, the evidence presented previously suggests that potentially infectious antigenic escape mutants with a similar fitness to wild-type HAV have been discharged into downstream water sources. However, the real biological impact of the mutations needs further investigation using in vitro growth competition and neutralisation assays.

Unique potentially infectious HAV IB strains have been discharged from WWTPs 1, 2, 4 and 5. There is a potential risk of HAV exposure to communities using water sources downstream these four WWTPs. Data suggest that the putative HAV mutants are circulating in the population serviced by WWTPs 1, 2, 4 and 5. Universal childhood vaccination is recommended for a region experiencing epidemiological shift [43]. Routine vaccination in the presence of environmentally stable antigenic escape variants could be ineffective and lead to their emergence and transmission within and between communities [42,44]. Therefore, in the community serviced by the four WWTPs, vaccination should be delayed until additional wastewater-based surveillance studies coupled with clinical studies can confirm or refute the circulation of these variants. Further surveillance of HAV strains is necessary to ensure the effective implementation of vaccine programmes.

## Figures and Tables

**Table 1:** Nucleotide sequences of primers used to amplify the VP1 and VP1/P2B genomic regions [10,35].

Genomic Region	PCR	Primer’s Name	Nucleotide Sequence (5′–3′)
VP1	First round	HAV1 (Forward)	gTT TTg CTC CTC TTT ATC ATg CTA Tg
		HAV2 (Reverse)	AgT CAC ACC TCT CCA ggA AAA CTT
	Second round	2172P (Forward)	gCT CCT CTT TAT CAT gCT ATg gAT
		3125N (Reverse)	CCT gCA TTC TAT ATg ACT CT
VP1/P2B	First round	2870P (Forward)	gAC AgA TTC TAC ATT Tgg ATT ggT
		3381N (Reverse)	CCA TTT CAA gAg TCC ACA CAC T
	Second round	2896P (Forward)	CTA TTC AgA TTg CAA ATA CAA T
		3289N (Reverse)	AAC TTC ATT ATT TCA TgC TCC T

**Table 2:** Hepatitis A virus (HAV) strains used during nucleotide sequence analysis.

Genotype	Strain Name	Geographical Location	GenBank Accession Number	Genomic Region	Source of Characterisation
IA	GBM	Germany	X75215	VP1, VP1/P2B	Clinical
IB	HM175	Australia	M14707	VP1, VP1/P2B	Clinical
IB	CFH-HAV	Sierra Leone	HQ246217	VP1, VP1/P2B	Clinical
IB	MBB	North Africa	M20273	VP1, VP1/P2B	Clinical
IB	HAF203	Brazil	AF268396	VP1, VP1/P2B	Unknown
IB	Banglane2000	Thailand	LC128713	VP1, VP1/P2B	Unknown
IB	HAV/Egy/BI-11/2015	Egypt	KX228694	VP1/P2B	Sewage
IB	L-A-1	China	AF314208	VP1, VP1/P2B	Unknown
IB	GP_66S-1, 67S-1, 106S-1, 109S-1, 110S-1, 111S-1, 112S-1, 112S2-1, 116S-1, 119S-1, 122S-1, 134S-1	Gauteng, SA	KJ433581 to KJ433592	VP1	Clinical
IB	GP_131S-1	Gauteng, SA	KJ207022	VP1	Clinical
IB	GP_PT66S, 67S, 106S, 109S, 110S, 111S, 112S, 112S2, 115S, 116S, 119S, 122S, 130S, 131S, 134S	Gauteng, SA	KJ492621 to KJ492635	VP1/P2B	Clinical
IB	GP_RV2-20120618-col1	Gauteng, SA	KJ492595	VP1	Surface water
IB	GP_11.1085-col7, 11.1085-col8, 11.1145-col1, 11.1145-col2	Gauteng, SA	KJ492598 to KJ492601	VP1	Wastewater discharge
IB	GP_RV2-20121112, RV2-20130318, K19-20130225	Gauteng, SA	KJ492654 to KJ492656	VP1/P2B	Surface water
IB	GP_11.1051, 11.1145, 11.1147, 11.1085	Gauteng, SA	KJ492657 to KJ492660	VP1/P2B	Wastewater discharge
IB	SZ_PT126S	Swaziland	KJ492643	VP1/P2B	Clinical
IB	SZ_PT29S-1, 126S-1	Swaziland	KJ433605 to KJ433606	VP1	Clinical
IB	K_KD-FEB, KD-NOV, KD-DEC, MB-JUL, NR-AUG	Kenya	KJ492671 to KJ492675	VP1/P2B	Surface water
IB	K_PT24S-1	Kenya	KJ433603	VP1	Clinical
IB	K_KD-DEC-1, KD-Feb-1	Kenya	KJ492607, KJ492608	VP1	Surface water
IB	T_PT34S-1	Tanzania	KJ433604	VP1	Clinical
IC	BCN70	Spain	HQ401240	VP1	Clinical
IC	Shellfish08-106	Spain	HQ401253	VP1	Food
IIA	CF53/Berne	France	AY644676	VP1, VP1/P2B	Clinical
IIB	SLF88	Sierra Leone	AY644670	VP1, VP1/P2B	Clinical
IIIA	SIM27	India	FJ227135	VP1, VP1/P2B	Clinical
IIIB	HA-JNG06-90F	Japan	AB258387	VP1, VP1/P2B	Clinical
IV	Cy145	Philippines	M59286	VP1, VP1/P2B	Simian
V	AGM-27	Kenya	D00924	VP1, VP1/P2B	Simian
V	KZN_Irr-20130530-1	KwaZulu-Natal, SA	KP875236	VP1	Surface water
V	KZN_Irr-20130530-2B	KwaZulu-Natal, SA	KP875241	VP1/P2B	Surface water

**Table 3:** Percentage of Hepatitis A virus (HAV) positive samples.

		Initial Screening	After Viability Treatment
Sewage	WWTP 1	10/11	8/9
	WWTP 2	11/12	8/8
	WWTP 3	5/12	1/5
	WWTP 4	7/7	6/7
	WWTP 5	10/12	7/8
Total		43/54 (80%)	30/37 (81%)
Treated wastewater discharge	WWTP 1	10/11	9/10
	WWTP 2	12/12	11/11
	WWTP 3	3/11	0/3
	WWTP 4	7/7	7/7
	WWTP 5	11/11	11/11
Total		43/52 (83%)	38/42 (90%)
Dam water		0/12 (0%)	−

**Table 4:** Hepatitis A virus (HAV) strains carrying amino acid change at the immunodominant and neutralisation epitopes.

		Name of Strain	^a,b^ 102	^a,b^ 171	^a,b^ 176	^c^ 217	^b^ 221
**WWTP 1**	^d^ R	DR4-1-2 MT721459	-	-	-	G217C	-
	R	DR5-1-F MT721466	-	-	-	-	K221E
**WWTP 2**	R	OR4-1-E MT721551	-	V171E	-	-	-
	^e^ E	OE7-1-302 MT721608	-	-	-	-	K221R
**WWTP 4**	R	FR6-1-B MT380571	-	-	A176T	-	-
	R	FR1-1-3 MT380587	S102P	-	-	-	-
	E	FE8-1-A MT380605	-	-	-	-	K221E
**WWTP 5**	R	VLR4-1-A MT721649	-	-	-	-	K221E
	R	VLR8-1-F MT721671	-	-	-	-	K221R
	E	VLE10-1-C MT721704	-	-	-	-	K221E
	E	VLE11-1-D MT721712	-	-	-	G217A	-

a: [11], b: [12], c: [15], d: R = sewage, e: E = treated wastewater discharge.

**Table 5:** In-frame deletion and amino acid changes recorded before and after viability treatment of a sewage (FR1) and treated wastewater discharge (FE8) samples.

		In-Frame Deletion		Amino Acid Changes	
		Before	After	Before	After
VP1	FR1	Two ^a^ aa deletion: position 241 to 242; FR1-1-2 (MT380586) and FR1-1-3 (MT380587) (Figure 6D)	Two ^a^ aa deletion: position 241 to 242; FR1-1v-a3 (MT380634) (Figure 6D)	S102P, R298K	S102P, G217D, R298K
	FE8	105 aa deletion: position 145 to 249; FE8-1-F (MT380609) on Figure 6B–D	−	V251T, V251I	V251I
VP1/P2B	FR1	−	−	R63K, R71S	R63K, C70S, R71S, M104I
	FE8	−	−	R63K, C70S, R71S, M104I	C70S, M104I

a: aa = amino acid.

## Supplementary Materials

The following are available online at https://www.mdpi.com/1999-4915/12/12/1468/s1, Supplementary file 1 of Figure 1: Fasta file of the sequence alignment used to infer phylogenetic analysis targeting the VP1 region of hepatitis A virus (HAV) strains detected in samples from all five wastewater treatment plants (WWTPs). Supplementary file 2 of Figure 3: Fasta file of the sequence alignment used to infer phylogenetic analysis targeting the VP1/P2B junction of hepatitis A virus (HAV) strains detected in samples from all five wastewater treatment plants (WWTPs). Supplementary file 3 of Figure 5: Graphic view of the complete protein alignment (from position 1 to 300) of the deduced amino acid sequences of the VP1 region of the HM175 strain and hepatitis A virus (HAV) strains carrying mutations at immunodominant and neutralisation epitopes. Supplementary file 4 of Figure 5: Fasta file of the sequence alignment of the VP1 region of the HM175 strain and hepatitis A virus (HAV) strains carrying mutations at immunodominant and neutralisation epitopes. Supplementary file 5 of Figure 6: Fasta file of the sequence alignment of the VP1 region of the HM175 strain and hepatitis A virus (HAV) strains carrying in-frame deletions. Supplementary file 6 of Figure 7: Graphic view of the amino acid sequence alignment of the VP1/P2B junction of the HM175 strain and 47 hepatitis A virus (HAV) strains carrying the C70S and/or M104I changes. Supplementary file 7 of Figure 7: Fasta file of the sequence alignment of the VP1/P2B junction of the HM175 strain and 47 hepatitis A virus (HAV) strains carrying the C70S and/or M104I changes.
