# Supplementary material for: Potentially Infectious Novel Hepatitis A Virus Strains Detected in Selected Treated Wastewater Discharge Sources, South Africa
Source: Viruses. 2020 Dec 19;12(12):1468. doi: 10.3390/v12121468 (PMC7765943; doi:10.3390/v12121468)
Supplement: Supplementary file 1 [file viruses-12-01468-s001.zip › viruses-979941-SI.pdf]

Supplementary file 1 of Figure 1: Fasta file of the sequence alignment used to infer phylogenetic analysis targeting the VP1 region of HAV strains detected in samples from all five WWTPs. Hepatitis A virus reference and closely related strains from GenBank are included in the alignment. Nucleotide sequence data of HAV strains previously characterised from clinical and water sources that were available in GenBank, were also added to the alignment. These strains were detected in specimens and samples collected from South Africa, Swaziland, Kenya and Tanzania.

Supplementary file 2 of Figure 3: Fasta file of the sequence alignment used to infer phylogenetic analysis targeting the VP1/P2B junction of HAV strains detected in samples from all five WWTPs. Hepatitis A virus reference and closely related strains from GenBank are included in the alignment. Nucleotide sequence data of HAV strains previously characterised from clinical and water sources that were available in GenBank, were also added to the alignment. These strains were detected in specimens and samples collected from South Africa, Swaziland, Kenya and Tanzania.

Supplementary file 3 of Figure 5: Graphic view of the complete protein alignment (from position 1 to 300) of the deduced amino acid sequences of the VP1 region of the HM175 strain and HAV strains carrying mutations at immunodominant and neutralisation epitopes. Conserved sites, substitutions and deletions are represented by dots, single-letter abbreviation and letter 'X', respectively. The red arrows points to the positions of the immunodominant (102, 171 and 176) and neutralisation (221) epitopes.

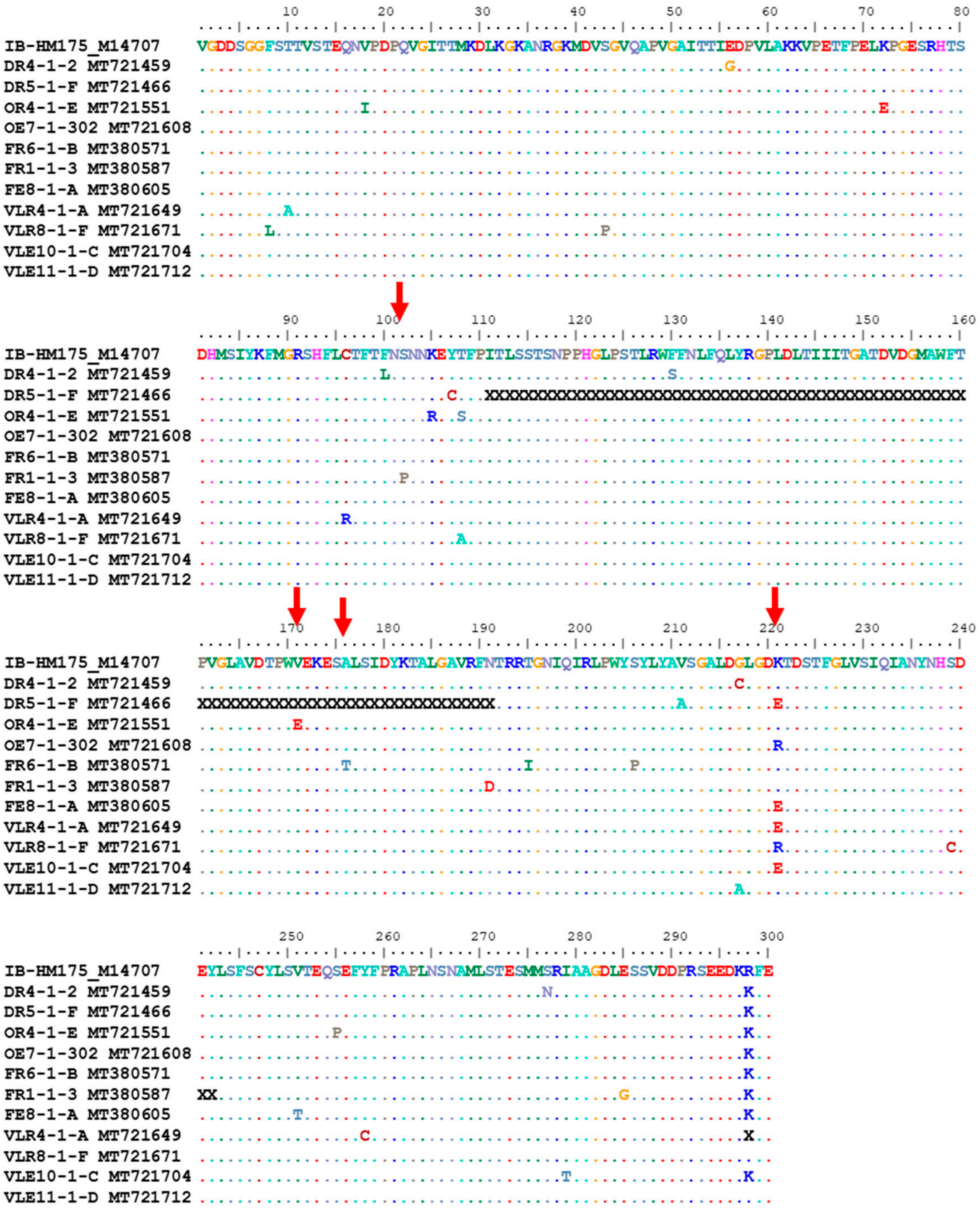

Supplementary file 4 of Figure 5: Fasta file of the sequence alignment of the VP1 region of the HM175 strain and HAV strains carrying mutations at immunodominant and neutralisation epitopes.

Supplementary file 5 of Figure 6: Fasta file of the sequence alignment of the VP1 region of the HM175 strain and HAV strains carrying in-frame deletions.

Supplementary file 6 of Figure 7: Graphic view of the amino acid sequence alignment of the VP1/P2B junction of the HM175 strain and 47 HAV strains carrying the C70S and/or M104I changes. Conserved sites, substitutions and deletions are represented by dots, single-letter abbreviation and letter ‘X’,

respectively. The red arrows and blocks highlight amino acid change at position 70 and 104, and, their surroundings, respectively. The black arrows point to HAV genotype II reference strains that carry the C70S change. The blue arrows point to HAV genotype III reference strains that carry the M104I change.

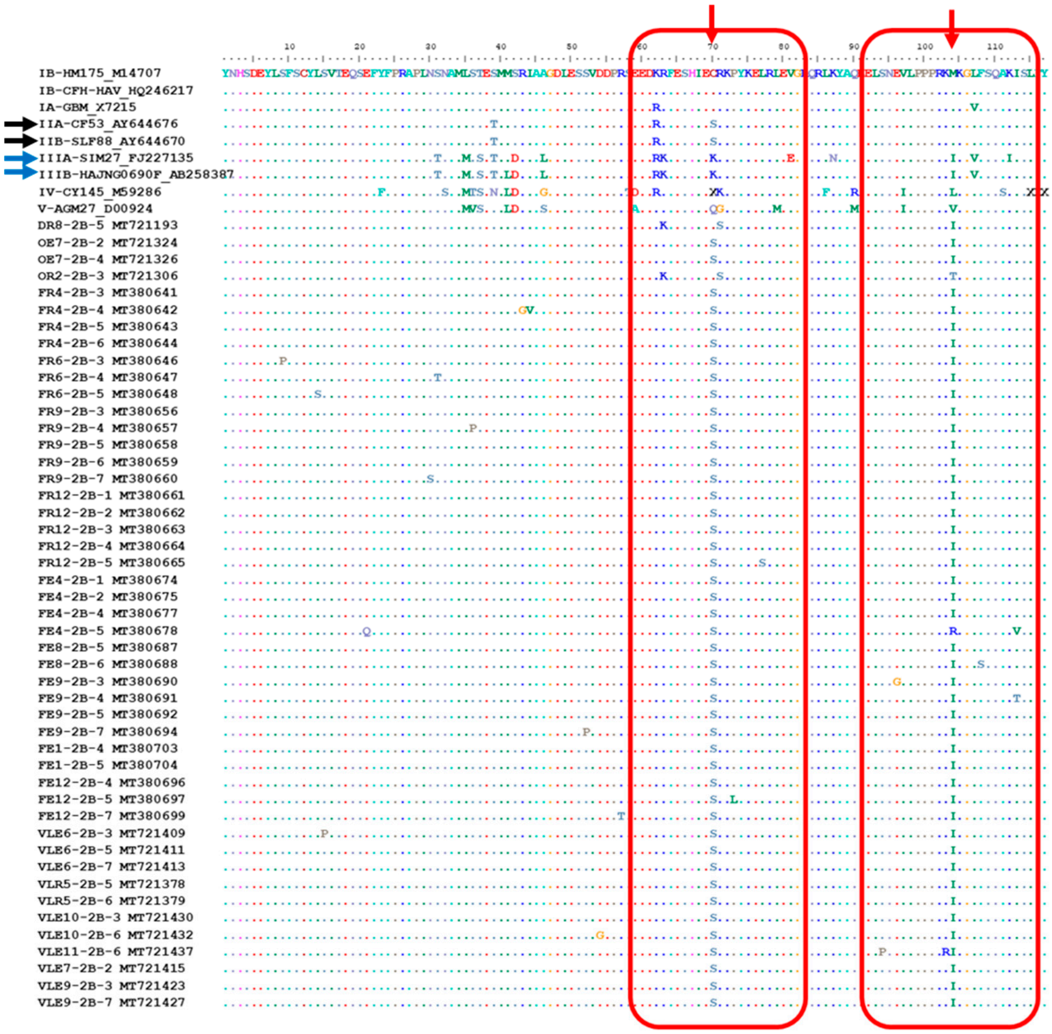

Supplementary file 7 of Figure 7: Fasta file of the sequence alignment of the VP1/P2B junction of the HM175 strain and 47 HAV strains carrying the C70S and/or M104I changes. The HAV genotype II and III reference strains are also included in the alignment.
